# The ties that bind: innovation configurations in low- and middle-income healthcare delivery settings

**DOI:** 10.1108/JHOM-09-2023-0275

**Published:** 2024-05-31

**Authors:** Wiljeana Jackson Glover, Sabrina JeanPierre Jacques, Rebecca Rosemé Obounou, Ernest Barthélemy, Wilnick Richard

**Affiliations:** Babson College, Wellesley, Massachusetts, USA; Wentworth Institute of Technology, Boston, Massachusetts, USA; OE Consulting, Raleigh, North Carolina, USA; SUNY Downstate Health Sciences University, New York City, New York, USA; Care 2 Communities, Cap-Haitien, Haiti

**Keywords:** Innovation management, Social innovation, Global health

## Abstract

**Purpose:**

This study examines innovation configurations (i.e. sets of product/service, social and business model innovations) and configuration linkages (i.e. factors that help to combine innovations) across six organizations as contingent upon organizational structure.

**Design/methodology/approach:**

Using semi-structured interviews and available public information, qualitative data were collected and examined using content analysis to characterize innovation configurations and linkages in three local/private organizations and three foreign-led/public-private partnerships in Repiblik Ayiti (Haiti).

**Findings:**

Organizations tend to combine product/service, social, and business model innovations simultaneously in locally founded private organizations and sequentially in foreign-based public-private partnerships. Linkages for simultaneous combination include limited external support, determined autonomy and shifting from a “beneficiary mindset,” and financial need identification. Sequential combination linkages include social need identification, community connections and flexibility.

**Research limitations/implications:**

The generalizability of our findings for this qualitative study is subject to additional quantitative studies to empirically test the suggested factors and to examine other health care organizations and countries.

**Practical implications:**

Locally led private organizations in low- and middle-income settings may benefit from considering how their innovations are in service to one another as they may have limited resources. Foreign based public-private partnerships may benefit from pacing their efforts alongside a broader set of stakeholders and ecosystem partners.

**Originality/value:**

This study is the first, to our knowledge, to examine how organizations combine sets of innovations, i.e. innovation configurations, in a healthcare setting and the first of any setting to examine innovation configuration linkages.

## Introduction

Innovation is generally defined as the initiation, implementation and/or adaptation of new products, services, processes, organizational structures, and technologies (Damanpour, 1991). In the health sector, innovations may aim to improve prevention, treatment, education, research, and/or well-being (Omachonu and Einspruch, 2010). Innovation is crucial for improving health outcomes in low- and middle-income settings (LMIS) (Mason *et al.*, 2015; Atun, 2012; Gardner *et al.*, 2007; Morel *et al.*, 2005; Sarkar and Mateus, 2022). In these complex emerging markets, the implementation of stand-alone innovations may be unsuccessful; instead, organizations must elegantly combine and carefully orchestrate many types of innovations (Doblin, 2015; Christensen *et al.*, 2019; Gardner *et al.*, 2007), i.e. *innovation configurations*.

The term *innovation configurations* (ICs) has been used to define interorganizational innovation *networks* at the regional or country level (Lafuente *et al.*, 2023), the *attributes* of an innovation, e.g. complexity, uncertainty, meaningfulness, and risk to observe differentiation between innovations (Ordanini *et al.*, 2014; Adams *et al.*, 2011) and the *types* or *categories* of innovation, e.g. service or marketization innovations (e.g. Moreira *et al.*, 2017; Miles *et al.*, 2017; Walker, 2008). We focus on the later definition to ICs to examine how organizations combine and orchestrate sets of innovation types in LMIS and thereby address two key gaps in the IC literature.

First, empirical studies of type-oriented ICs tend to focus on marketing, product/service, clinical, technological (i.e. digital health), and organizational structure-related innovations, perhaps in part because they have primarily been conducted in high-income settings with relatively stable business models and social services given institutional norms (e.g. García-Rayado and Callens, 2024; Thijssen *et al.*, 2023; Moreira *et al.*, 2017). However, healthcare settings also require social innovations (Poblete *et al.*, 2023) and business model innovations (Šlapáková Losová and Dvouletý, 2024; Furnival *et al.*, 2019), particularly in LMIS (Christensen *et al.*, 2019; Gardner *et al.*, 2007; Mason *et al.*, 2015). Conceptual studies suggest that social innovations may ensure the maximum uptake of product and service innovations (Gardner *et al.*, 2007) while business model innovations may create more stability and infrastructure as well as generate new positions in underserved markets (Christensen *et al.*, 2019; Gardner *et al.*, 2007; Mason *et al.*, 2015). Thus, further research is needed to understand how product/service, social, and business model innovations are combined in LMIS.

Second, it is also unclear what links innovations together and the conditions under which various sets of innovations emerge. Accounting for the mechanisms that support IC is particularly relevant in the context of healthcare. Innovation in complex sectors like health cannot be mechanistically achieved, but is influenced by the emergent organizational behaviors and agents’ characteristics (Glover *et al.*, 2020; Plsek and Wilson, 2001). Previous IC studies have not included these linking factors but call for future research to examine the conditions under which various sets of innovations are needed (Walker, 2008).

Thus, this study focuses on two key questions for empirical examination: (1) What factors link different innovation types, i.e. product/service, social, and business model innovations, to form successful ICs? and (2) How do the conditions under which organizations should implement ICs influence the innovation types and linkages? We adopt configuration theorizing (Furnari *et al.*, 2021) to examine how multiple innovation types combine via a novel concept that we introduce, *IC linkages*. We anticipate that need and opportunity identification as well as characteristics of organizational members may serve as the “ties that bind” ICs. Second, we leverage a contingency approach to examine the extent to which the nature of the organization, i.e. locally led private healthcare organizations vs foreign-based public-private partnerships, influences the IC.

In summary, this study assesses the factors that link multiple innovation types, i.e. IC linkages, as well as identify the conditions under which different ICs and linkages emerge in healthcare organizations. We use a qualitative approach via semi-structured interviews and supporting secondary sources with six healthcare organizations (three local/private and three foreign-led/public-private partnerships) in *Repiblik Ayiti* (Haiti). We discuss our contributions to configuration theory by describing the linkages that combine innovation types. We discuss our contributions to the healthcare innovation literature by describing the conditions under which different ICs may be ideal.

## Background

### ICs: combining innovation types

Our current inquiry focuses on combining *types* or *categories* of innovation (e.g. Moreira *et al.*, 2017; Miles *et al.*, 2017; Walker, 2008). In some sense, all innovation is the introduction of new combinations (Hagedoorn, 1996). We focus on product/service innovations, social innovations, and business model innovations as they are central to the IC literature (Walker, 2008) and the advancement of healthcare innovation in LMIS (e.g. Mason *et al.*, 2015; Gardner *et al.*, 2007; Atun, 2012), but have had limited empirical inquiry to date. Furthermore, examining how innovation types may be combined contributes to configuration theory, or why attributes combine in complex, and at times contradictory ways (Furnari *et al.*, 2021). Our next section describes these three types, followed by a review of the linkages that may lead to different configurations.

### Innovation types: product/service, social, and business model innovations

Product and service innovations include the set of core and complementary offerings by an organization, their features, and functionality (Doblin, 2015). Recent product and service innovations in LMIS in the U.S. include increased remote monitoring, digital and telehealth solutions, device and treatment advancements, and integrated community health center care (García-Rayado and Callens, 2023; Thijssen *et al.*, 2023; Moreira *et al.*, 2017; Lewis *et al.*, 2014). Product and service innovation capacity may enhance LMIS health system capabilities (Atun, 2012; Morel *et al.*, 2005; Gardner *et al.*, 2007).

Product/service innovations are a necessary but insufficient condition for the improvement of efficiency and equity in healthcare delivery systems (Gardner *et al.*, 2007). Individual product/service innovations may lead to a “silver bullet” mindset towards innovation (Rye and Kimberly, 2007). Health systems increasingly seek to decrease health inequities caused by upstream influences, including the socioeconomic, political and cultural context and daily living conditions (Mason *et al.*, 2015; Gardner *et al.*, 2007). Increasing environmental uncertainty and resource constraints also influence innovation in healthcare organizations beyond traditional medical decision-making boundaries, including addressing social risks (Rye and Kimberly, 2007). Thus, we include social innovation within our examination of ICs.

Social innovations are solutions to social problems that are more just, effective, efficient, and/or sustainable than existing solutions and accrue value to society as a whole (Phills *et al.*, 2008). In healthcare, social innovations can include co-creation of support and welfare activities with community members with lived experiences (e.g. cancer survivors as described by Poblete *et al.*, 2023), free care models for marginalized populations (while still charging a fee for others) as well as solutions that address social determinants of health or SDOH (Mason *et al.*, 2015), including economic stability, neighborhood and physical environment, education, food, and community and social context (Artiga and Hinton, 2018). Some scholars suggest that addressing social needs is outside of the purview of medical professionals (Rubin, 2019). However, others propose that healthcare delivery systems need social innovations to ensure the maximum uptake of product and service innovations and for sustainable improvement of health system effectiveness and efficiency because they help to adapt new products to local conditions (Gardner *et al.*, 2007).

Business model innovations describe how the profit structure, i.e. the margin between the assets and fixed cost structure, supports the value proposition, i.e. the product or service that helps customers more effectively, conveniently, and affordably (Hwang and Christensen, 2008). Business model innovations relate to process-oriented management innovations (Øvretveit *et al.*, 2012), but with the key difference that business model innovations incorporate revenue models that directly impact the financial outcomes of the organization. Business model innovations in healthcare are relatively understudied compared to product and service innovations (Rye and Kimberly, 2007), but are becoming more commonly discussed due to limitations of publicly-funded services. Examples include charging fees for services (Hearld *et al.*, 2018), implementing subscription and consultation fees (Šlapáková Losová and Dvouletý, 2024), creating multiple revenue streams (Poblete *et al.*, 2023), and can involve incorporating interorganizational partnerships to secure funding (Šlapáková Losová and Dvouletý, 2024; Wankah *et al.*, 2022; Poblete *et al.*, 2023; Hearld *et al.*, 2018). Business model innovations help to ensure that strategic choices and associated investments are made towards emerging opportunities, particularly in LMIS (Best *et al.*, 2018; Christensen *et al.*, 2019). Stand-alone product/service or social innovations may fail in LMIS due to limited financial infrastructure and sustainability (Smith *et al.*, 2021; Gardner *et al.*, 2007; Hwang and Christensen, 2008). Thus, we anticipate that business model innovations will accompany successful product/service and social innovations.

### IC linkages

Next, we explore how innovation types combine via configuration theorizing. Configurational theorizing involves scoping plausible coherence among a constellation of attributes (in our case, innovation types), linking them via their interdependences, and naming the configurations holistically to their orchestrating themes (Furnari *et al.*, 2021). In general, when an organization implements a larger number and variety of innovations, it positively impacts success (Damanpour, 1991). Combining and implementing different innovation types requires linkages across internal and external boundaries, bringing additional complexity (Doblin, 2015). The emergent organizational behaviors and agents’ characteristics under complexity may influence innovation (Glover *et al.*, 2020; Plsek and Wilson, 2001). We extend these studies to examine how they may also link ICs.

Organizational behaviors can serve as IC linkages. Examples include need and opportunity identification behaviors such as sense making, boundary scanning and searching within the organization, the communities it serves, and the broader health ecosystem for unsolved issues, patient needs, and ideas (Furnival *et al.*, 2019; Tan *et al.*, 2005; Glandon and Counte, 1995). These behaviors may be more likely in settings where healthcare staff are “attracted” to innovations that connect clinical service and social needs, e.g. providing services to underserved patients (Glover *et al.*, 2020; Plsek and Wilson, 2001). Need and opportunity identification behaviors may also connect service and business model innovations. For example, when organizational members identify new markets for whom existing products were inaccessible due to cost, they create more affordable or accessible innovations (Christensen *et al.*, 2019; Bosa, 2008). In other words, they combine product/service, social, and business model innovations through need identification. Thus, we anticipate that these need and opportunity identification behaviors serve as linkages between innovation types.

Organizational characteristics may also serve as IC linkages. While the health organizations exist within heavily regulated ecosystems may stifle innovation, there are also characteristics including autonomy, knowledge, and environmental understanding that may inspire organizational members to innovate despite these perceived constraints. For example, autonomy can enable innovation as organizational members learn new things and discover new opportunities (Furnival *et al.*, 2019; Puranam *et al.*, 2006; Jansen *et al.*, 2006). Organizational knowledge includes technical knowledge of the innovations within the configuration. For example, a qualitative study of two organizations in Germany found that deep knowledge of patient-centered care models and emerging financial models encouraged entrepreneurial physicians to form group practices with new fee structures (e.g. Bosa, 2008). Organizational members with environmental understanding of other stakeholders may be more successful at implementing innovations via relationship building to achieve value alignment with ecosystem partners (Wankah *et al.*, 2022), particularly in LMIS (Bloom and Dees, 2008).

### The context for ICs

Finally, we leverage a contingency approach to examine how the nature of the organization impacts the IC. Configurations of service, social, and business model innovation types have been examined in public organizations (Walker, 2008). Walker (2008) calls for future research to examine ICs via contingency theory. Thus, we examine ICs in healthcare organizational structures beyond the public model. Healthcare organizations have several structural variations, three of the most common being public organizations (run fully by the government), private organizations (with no direct government involvement) and public-private partnerships (combining private and state-run efforts). While fully public healthcare may be more common in high-income settings (e.g. the U.K.), public funding is declining in LMIS (World Health Organization, 2018; Alfonso *et al.*, 2021). Therefore, we focus on innovation combinations in private organizations and public-private partnerships. Public-private organizations may primarily function as not-for-profit while still exploring some revenue generating activities, thereby influencing the product/service, business model, and service innovations chosen. Private organizations may primarily function as for-profit but with a not-for-profit arm to incorporate social innovations. We explore how these structural choices may influence the chosen IC.

In addition to the delineation between public-private partnerships and private organizations in LMIS, we also examine the ICs of local and foreign organizations. In recent years, foreign organizations increasingly partnered with public institutions to provide goods and services and create jobs. This approach can support local efforts but is still distinct from the efforts of local individuals who start businesses and organizations. Thus, we compare locally led private organizations to foreign-based public-private organizations. We do not suggest that one approach is preferred. Rather, we examine the extent to which ICs and their linkages are contingent upon the organization being a local vs foreign organization. We recognize that other organizational structures (local public organizations, foreign private organizations) exist. However, because innovations in local public organizations are more often studied and purely private foreign organizations are becoming less common in LMIS, we focus our efforts on the growing, yet understudied organizational structures (locally-led private organizations vs foreign-based public-private organizations). We also note that over time, at least one of our locally led private organizations engaged in a public-private partnership. Thus, our definition of locally led private organizations focuses on their organizational genesis.

## Methods

### Research approach, setting, and team

We used a qualitative research design to examine ICs and their linkages within healthcare organizations in Haiti. Qualitative methods are effective to help develop theory on topics with limited research (Miles and Huberman, 1994), as is the case of ICs. We leverage directed content analysis to synthesize data from participating organizations and to examine the commonalities and general structure of their ICs and linkages.

We used the COnsolidated criteria for REporting Qualitative research (COREQ) checklist (Tong *et al.*, 2007) to report our findings. We begin with the research setting and team. Haiti’s healthcare delivery system is a mix of public, private, and public-private organizations (Barthélemy *et al.*, 2019; Peck *et al.*, 2019). Publicly, the Ministry of Health is named Ministère de la Santé Publique et de la Population (MSPP). Privatization of healthcare in Haiti may be further divided into locally-based private entities, and internationally-based private entities, with the latter including collaborations between the two different modes of privatization. Much of healthcare is increasingly privatized by internationally-based entities, particularly after the 2010 earthquake. This expansion of privatization by foreign non-governmental organizations (NGOs) temporarily increased care capacity, but with decreased funding available to the NGOs, healthcare delivery and public services in general are now a patchwork system with limited required accountability to and integration with the public system. The need for increased healthcare capacity while simultaneously addressing social needs in a financially sustainable manner makes Haiti an ideal place to examine the IC linkages.

Our research team included two M.D. clinicians with ∼30 combined years of clinical experience (one in Haiti and one the U.S.) who provided interview contacts, regional context, interview guide feedback, and reviewed the analysis and overall manuscript for contextual accuracy. Our two Ph.D. researchers with ∼25 combined years of qualitative research experience in health settings drafted the interview questions, conducted interviews with two of the six organizations, sent follow-up questions and confirmations via email, and coded and analyzed the data. Our MBA academic researcher with 9 years of social innovation experience in Haiti provided interview contacts, conducted interviews and sent follow-up questions and confirmations via email to four of the six organizations. Four of the five researchers spoke Haitian Creole as their first language and one spoke English.

### Sample

For this study, we conducted individual and small group interviews with three foreign-based organizations (8 interviewees) and three locally-led organizations (6 interviewees). Our overall sample of 14 interviewees aligns with field methods best practices that find saturation occurs between six and twelve interviewees (Guest *et al.*, 2006). Across the six locations, we include organizations that serve most catchment areas including Southern Haiti, Northern Haiti and Côte des Arcardins, Central Plateau of Haiti and the capital, Port-au-Prince. Organizations were recruited and selected using a non-random method: contacts made through research colleagues and professional and conference networks. No requested organizations declined to participate. Selected organizations were provided with consent forms and the interview guide via email in advance of the interview. During each interview, one to four employees from each organization were in attendance. We discuss the combination of individual and focus group interviews in more detail in our limitations. Interviewees included organization founders, C-suite leadership, administrators, physicians, and program directors. Table 1 presents a brief description of each organization and participants. Interviews for four organizations occurred in person at their clinic offices and interviews for two organizations occurred via Zoom. The study was approved both by a U.S. academic Institutional Review Board and the National Bioethics Committee of Haiti’s Ministry of Public Health.

Specific questions within our semi-structured interview guide included:

1. How did you decide to be for profit vs not for profit? Tell us your rationale.
2. What are all of the services you provide? Why did you decide to provide them? List all products/services, e.g. healthcare, financial, social, etc.
3. What does innovation look like in your organization?
4. What influenced your business model?

As they responded to these questions, we asked interviewees to reflect broadly on their innovation journeys since their founding or start with the organization; we did not bound the question to one specific innovation or time period. Interviews lasted for 60–150 min, and all interviews were taped and transcribed. Responses in both Haitian Creole and English were recorded, translated, and transcribed into field notes from all meetings. Follow-up questions were asked via email. These follow up questions enabled us to revise and improve the preliminary conclusions from the data. We also used available public information (e.g. websites and annual reports) as additional data sources.

## Analysis

The qualitative data was analyzed via manual coding. To code the response segments, we used an directed content analysis approach. Directed content analysis is ideal in cases where some theory exists that would benefit from further description and can provide some predictions about the variables of interest, helping to determine an initial coding scheme (Hsieh and Shannon, 2005). We began with a deductive list of primary themes summarized in Table 2. For each theme, examples formed the individual codes. These individual codes emerged inductively. To determine analyses’ accuracy and consistency, one researcher read the transcripts separately and completed sequential rounds of coding. Then, another researcher reviewed the transcripts and resolved discrepancies in coding. Emerging patterns within the data were agreed upon via group discussions with the research team (Miles and Huberman, 1994).

## Findings

### ICs

Table 3 presents a summary of the product/service, social, and business model innovations across sites as well as illustrative quotes for each category. We provide examples of each category and then examine their intersections.

*Product/service innovation.* Most reported service innovations aimed to increase access to offerings that were previously unavailable or not proximal to certain communities. Organizations A, B, C, and F focused on extending services via building infrastructure in underserved, rural regions of the country. Many of the structures themselves were innovative in terms of their use of solar power and sustainable materials. Organization D achieved greater service proximity via deploying doctors within manufacturing facilities and neighborhood clinics within larger cities. All organizations reported new clinical care offerings to their region, including surgical procedures and additional obstetric care.

*Social innovation*. The social innovations across the participating organizations were aimed to increase access to care and address SDOH. For example, Organization D focused on community-based hiring and focusing their insurance sales and service advertising at social institutions including churches and schools. Organization F leveraged community engagement and community-focused training and hiring to identify and launch initiatives in education, microcredit, and sanitation. As the only for-profit organization in our sample, Organization E began a foundation to offer free or discounted care. Organization C invested in the expansion of free services at additional public-owned primary care centers. Organizations A and B also launched complementary services to address SDOH. Organization A invested solar power and discussed ideas to sell surplus energy to local and neighboring municipalities. Organization A also hosted a vocational school, an agricultural and nutrition organization that produces meals for hospital, manufactures fortified peanut butter for malnourished children, and a community garden. Organization B invested in agriculture, through the launch of a poultry program, local farming support, and a weekly market. They also started a water program, describing it as a potential financially sustainable model over time.

*Business model innovation*. We found three dominant approaches to business models across our participating organizations. The first (by Organizations A and B) focused on providing free care and charging a small fee for registration. Additional costs are primarily funded via philanthropy. Organization C employed a governmental partnership model that included a combination of governmental funding, philanthropic donations, and earned revenue. This model allows Organization C to operate both private and public clinics. Organizations D, E and F took more revenue-generating focused approaches. Organization E charges a direct-to-consumer fee and was one of the first for-profit healthcare corporations in the country. Organization D is a Haitian-owned non-profit network of hospitals and clinics, offering a variety of direct-to-consumer and employer-paid insurance models. Organization F also charges a direct-to-consumer fee and incorporates a microcredit offering that generates revenue for the organization as well as funds a community fund that supports referral care.

### Configurations formed through sequential and simultaneous combination processes and linkages

**Figure 1:**
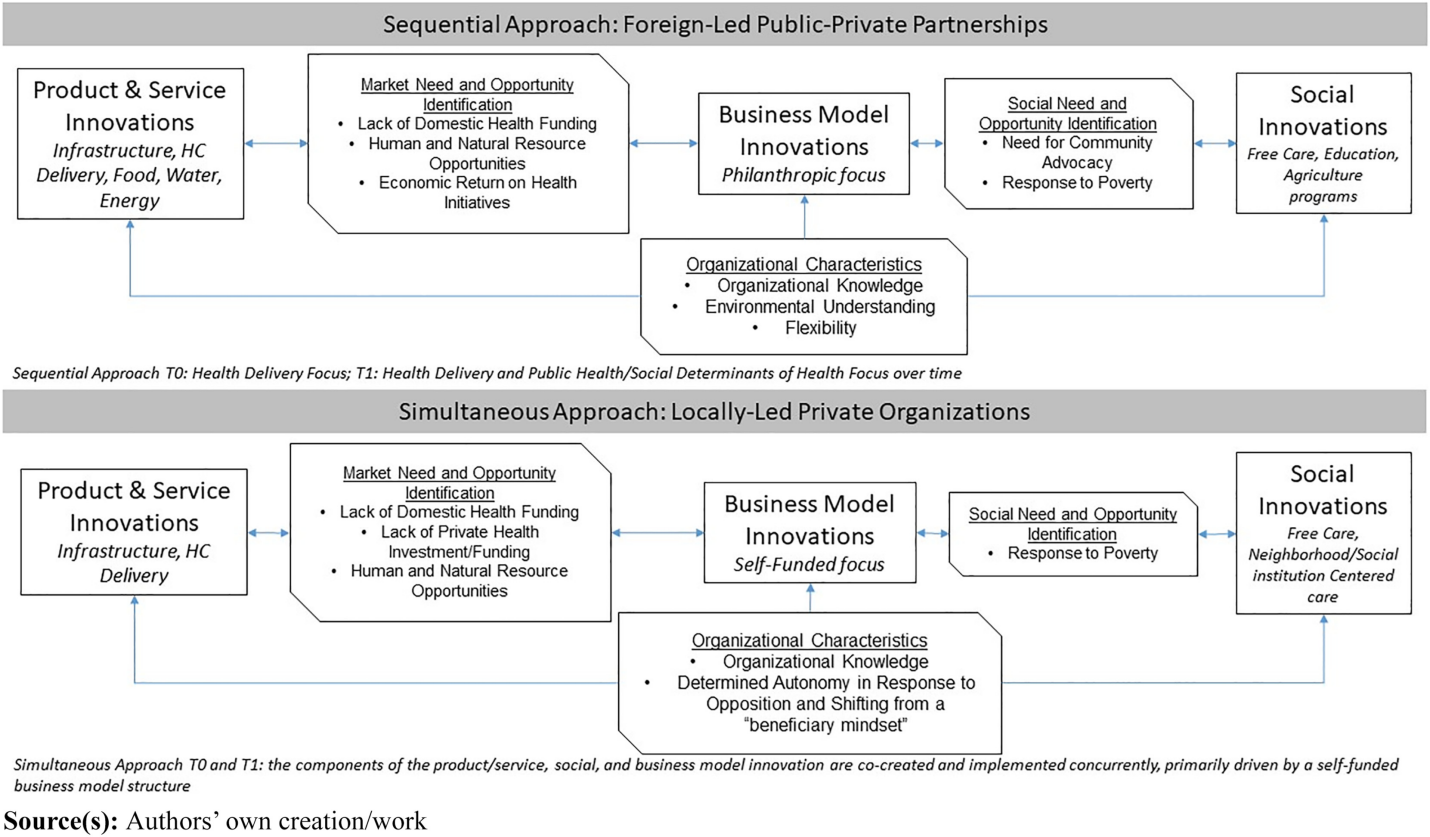
A simultaneous and sequential approach to combining healthcare innovations

Now we examine the two dominant configuration approaches and their IC linkages (Figure 1): a sequential configuration process and a simultaneous configuration process. Among our participants, we found that organizational context influenced configuration choice. We also found that the configuration process influenced the IC linkages amongst participants.

*Sequential configuration process and linkages.* First, we found a *sequential configuration process* where the product/service, social, and business model innovations are created and implemented over time, driven by a primarily philanthropic business model. Sequential configuration processes were used by the foreign-led public-private participating organizations (Organization A, B, and C) and expanded to a broader set of innovations over time, particularly innovations that addressed broader public health and SDOH needs. Organization C was the most embedded example of a public-private partnership with the organization directly running public clinics as well as a set of private clinics by combining government, philanthropic, and earned revenue sources to provide both free and fee-for-service care. This approach was executed over time, with Organization C beginning with private clinics (Product/Service Innovation), then financially supporting public clinics (Social Innovation) and eventually running public and private clinics (Product/Service, Social, and Business Model Innovation). Organizations A and B do not directly run public clinics, but engage in extensive state collaboration to develop and execute their innovations and primarily offer free services. They both began with clinics that became a larger network of hospitals and clinics (Product/Service Innovation). Over time, they expanded their scope to include public health offerings like therapeutic foods and water and energy to run the hospitals (Product/Service Innovation) as well as community-based offerings to include energy, water, agricultural, and educational programs (Social Innovations).

Sequential IC linkages related to Market Need and Opportunity Identification among Organizations A, B, and C were a *lack of domestic health funding*, *human and natural resource opportunities*, and the *economic return of health initiatives*. Sequential ICs were also linked by Social Need and Opportunity Identification, specifically the *need for community advocacy* and *addressing underlying poverty*. Community advocacy includes building relationships with village members as well as governmental partners. These Social Need and Opportunity Identification linkages may also represent intrinsic drivers for organizational members, e.g. pride in solving a problem or identifying a solution as their idea, which is often associated with addressing social ills (Pype *et al.*, 2018).

Finally, IC linkages related to Organizational Characteristics among Organizations A, B, and C included *organizational knowledge*, *environmental understanding of the importance of partnerships*, and *organizational flexibility*; these characteristics further supported a sequential configuration process. For example, Organization A cited their ability to provide more complementary Social Innovations because of having a staff with both public health and agricultural backgrounds.

*Simultaneous configuration process and linkages.* We also found a *simultaneous configuration process* within Organizations D, E, and F where the components of the product/service, social, and business model innovation are co-created and implemented concurrently, primarily driven by a self-funded business model structure. While they scaled over time, they tended to incorporate social innovation within their business models. Organization D began its insurance offerings and care provision via clinics and doctor worksite visits around the same time. Organization E launched itself as a for-profit practice (the first healthcare corporation in the country) with five founding physicians, its focus on cooperatively financing the latest equipment and providing cutting-edge ENT services, and provided a pool of funds for complementary and discounted care concurrently. This pool of funds eventually became its non-for-profit foundation. Through a series of community engagement meetings, Organization F simultaneously choose its core initiatives (health and education) and charged for medical services and tuition.

Simultaneous IC linkages related to Market Need and Opportunity Identification among Organizations D, E, and F were a *lack of domestic health funding* and *lack of private health funding*. While Organizations D, E, and F also observed a lack of state investment in healthcare at their inception, they also reported difficulty securing external financial support from larger commercial or NGO sources when they started. Thus, these organizations began as self-financed ventures with some smaller external donations. These organizations eventually found larger external supporters and state funding after proof of concept of their models, but this lack of initial external support may have contributed to these organizations taking a simultaneous configuration approach.

These organizations focused less on Social Need and Opportunity Identification linkages, though they did respond to poverty via free care, neighborhood-centered care initiatives, and were driven by a need for community empowerment. This could be in part because of their embeddedness in the setting, i.e. local innovators are so aware of social needs, that the social needs themselves may not drive innovative action. Instead, we see a stronger focus on Organizational Characteristic linkages among these locally-led private organizations. *Determined autonomy through external opposition* and *shifting from a “beneficiary mindset”* were unique factors for these locally-led private organizations. For example, Organization E participants reported being told that their organization would not last as a private for-profit cooperative. There was an instance where Organization F would not engage foreign partners unless they valued community empowerment as opposed to individual child sponsorship. The combination of the lack of private health funding and this determined autonomy appears to drive unique business models among these organizations that were new to the country at the time, e.g. insurance models and cooperative financing.

## Discussion

This study examined ICs and IC linkages as contingent upon organizational structure via configuration and contingency theorizing (Furnari *et al.*, 2021; van de Ven *et al.*, 2013). The following develops the theoretical and practical implications of (1) the identified ICs and (2) the IC linkages across our research participants. Given our qualitative methodology, future quantitative research should further examine these implications.

First, the ICs literature suggests that combining types of innovations may ensure maximum innovation adoption and increase more organizational stability and infrastructure (Christensen *et al.*, 2019; Gardner *et al.*, 2007). Studies that do not explicitly seek to combine innovations also suggest that healthcare settings benefit from incorporating social and business model innovations with their rom not new product and service innovations (e.g. digital health applications or new clinical device technologies) (Poblete *et al.*, 2023; Šlapáková Losová and Dvouletý, 2024; Furnival *et al.*, 2019). Our study contributes to this literature by empirically examining the combined use of product/service innovations, social innovations, and business model innovations within our setting, which were not previously the focus of empirical IC studies in healthcare (e.g. Moreira *et al.*, 2017; Miles *et al.*, 2017). We observed that all six organizations pursued product/service, social, and business model innovations. Our participating healthcare organizations in Haiti are providing new services in part because they are also experimenting with different business models (e.g. insurance models, fee structures based on ability to pay), and are considering how to commercialize social innovations (e.g. water, education, and energy) for financial sustainability. Theoretically, these findings support a configurational approach (Furnari *et al.*, 2021) to future innovation inquiry, as it is not necessarily the case that the product/service innovations “caused” the business model or social innovations, but rather that they are connected, synergistic types of innovation that help each other succeed. Future quantitative research could further examine their relationships in concert. Practically, these findings provide intraorganizational approaches that can support private healthcare organizations and alleviate the burden on public healthcare finances, adding to the interorganizational approaches explored byŠlapáková Losová and Dvouletý (2024).

Second, via configuration theorizing (Furnari *et al.*, 2021) we also identify IC linkages. The ICs literature to date does not examine linkages between innovation types (Moreira *et al.*, 2017; Miles *et al.*, 2017). Thus, we contribute by identifying some common and differentiating IC linkages within our setting, depending on the organizational structure. We discuss the practical and theoretical implications of these linkages.

Common IC linkages across all six organizations included two Market Need and Opportunity Identification links (*a lack of domestic health funding* and *human and natural resource opportunities*), one Social Need and Opportunity Identification link (*responding to poverty*), and one Organizational Characteristic link (*organizational knowledge*). As noted in Figure 1, these Market Need and Opportunity factors tended to be mentioned with, or “link” the relationship between product/service innovations and business model innovations. The Social Need and Opportunity Identification links may connect the relationship between social innovations and business model innovations. Organizational Characteristics provide additional linkages throughout a given configuration. Examining these complementarities allows us to also explore how these linkages might enhance the mutual relationship between innovation types. The emergence of *a lack of domestic health funding* from our qualitative data suggests that innovations from private organizations are needed to fill gaps in publicly funded health systems (Šlapáková Losová and Dvouletý, 2024; Christensen *et al.*, 2019). *Recognizing human and natural resource opportunities* is an important sensing capability to support innovation and entrepreneurship (Furnival *et al.*, 2019; Bosa, 2008). Identifying it as a linkage within ICs suggests that the recognition of such resources may also enhance the relationship between the value created by a product/service innovation and the business model needed to bring that value to the market. Practically, organizations in LMIS may wish to encourage and train leaders to identify such resources when public funding is insufficient.

The linkage *responding to poverty* relates to the social innovation literature (e.g. Artiga and Hinton, 2018). Its identification as a linkage contributes to the broader innovation literature and may help organizations to avoid developing social innovations in a silo, which has been critiqued as unsustainable (Smith *et al.*, 2021; Gardner *et al.*, 2007; Hwang and Christensen, 2008). Finally, the linkage *organizational knowledge* was common across all six organizations. Innovation advances skills and know-how among innovation collaborators (Šlapáková Losová and Dvouletý, 2024). Our findings extend this work, suggesting that organizational knowledge may also connect the ICs among our research participants, influencing the particular innovations chosen for implementation.

Differentiating IC linkages also emerged among our research participating organizations based on differences in organizational structure. Among the simultaneously configured innovations across the three locally led private organizations, one differentiating Market Need and Opportunity Identification link (*a lack of private health investment/funding*) and two differentiating Organizational Characteristic links (*determined autonomy in response to opposition* and *shifting from a “beneficiary mindset”*) emerged. Collectively, these factors contribute to previous calls for research to examine how frugal service innovations may yield profits in LMIS to achieve financial sustainability (Sarkar and Mateus, 2022). Our participating organizations D, E, and F appeared to respond to challenges raising capital by leveraging their determination in response to opposition as intrinsic motivation to develop holistic, revenue generating ICs. This aligns with previous studies that suggest healthcare staff are “attracted” to innovations that connect with their intrinsic motivations (Glover *et al.*, 2020; Plsek and Wilson, 2001) and that contextual resistance can be leveraged as opportunity for innovation (Newth and Woods, 2014). Practically, these findings suggest that locally led private organizations in LMIS can choose to appreciate the ways in which their innovations can evolve and ultimately succeed because of opposition (Newth and Woods, 2014). Future research and policy should also consider the scaling capabilities of such locally led private organizations if barriers to funding were not present and work to eliminate such barriers to entry.

Among the sequentially configured innovations across the three foreign based public-private organizations, one differentiating Market Need and Opportunity Identification link (*economic return on health initiatives*) and two differentiating Organizational Characteristic links (*environmental understanding of the importance of partnerships* and *flexibility*) emerged. Collectively these findings contribute to the healthcare innovation literature, specifically studies that focus on collaborative, interorganizational approaches to innovation (e.g. Šlapáková Losová and Dvouletý, 2024; Wankah *et al.*, 2022; Poblete *et al.*, 2023; Hearld *et al.*, 2018). Our participating foreign-based public-private partnerships appear to benefit from pacing their efforts alongside additional ecosystem partners. Pacing and time are not explicitly included in these previous studies and implies that there may be an iterative, nonlinear process to sensing and linking configurations (Furnari *et al.*, 2021) particularly when other organizations are involved. Organizations A, B, and C also appear to contribute to their partnerships via their ability to describe, and at times quantify, the economic return of their health initiatives to their public partners, e.g. increase in days worked among population of a surgical innovation is introduced. Because health product/service innovations are at times critiqued for lacking evidence of impact (Thijssen *et al.*, 2023), this linkage demonstrates a practical approach to strengthen the connection between a product/service and business model innovation. Being able to describe the economic return of a health initiative may also help to attract the additional finances that are often needed in healthcare innovation (Šlapáková Losová and Dvouletý, 2024).

## Limitations

We acknowledge that, as a qualitative study, our findings represent ICs and IC linkages within the organizations participating in this research. Our findings do not represent all ICs and linkages within healthcare organizations in LMIS. The generalizability of our findings is subject to additional quantitative studies to empirically test the suggested factors and to apply it to other health care organizations and countries. Additional factors that may limit the relevance of the findings beyond the participating organizations include ecosystem and cultural factors. Future research could also consider research methodologies that have been adapted to LMIS contexts, e.g. the recent adaptation of the Consolidated Framework for Implementation Research to Low-and Middle-Income Countries to account for ecosystem factors (Means *et al.,* 2020). Moreover, in this study, we identified ICs and linkages from the perspective of healthcare organizational leaders and clinicians. Future studies should explore these ICs and linkages from a patient perspective (Šlapáková Losová and Dvouletý, 2024; Berwick, 2009).

There were also sampling limitations. Our sample size of interview participants, particularly for the locally led private organizations, was smaller, in part because this organizational form is relatively rare in the country and the organizations tended to be smaller. As more locally led private organizations emerge in LMISs, their practices should be further examined beyond this study. Due to scheduling limitations, we interviewed some participants in focus groups and some participants individually. The hierarchy between focus group interviewees may influence respondents’ statements, which presents a potential limitation. We aimed to mitigate this limitation by following up via email individually with participants with a copy of their interview transcripts, asking if they had any additional comments or concerns. This limitation was also an opportunity to observe the convergence of responses across focus groups and the individual interviews which can indicate enhanced trustworthiness in findings (Lambert and Loiselle, 2008). Responses, whether conducted one on one or as a focus group, reflected similar themes.

Finally, our data were collected prior to the most recent 2024 political uprisings in Haiti. While many health systems in the capital have ceased operations due to gang violence, some of our participating organizations outside of the city center and in the countryside remain operable. We believe their ICs and linkages observed prior to these uprisings are still important for the IC body of knowledge as they provide examples of new practices that, through future research and practice, may inform ICs in other LMIS.

## Conclusions

We introduce the concept of IC linkages and suggest a more synergistic approach of how private healthcare organizations and public-private healthcare partnerships can combine innovations. Given the rising focus on improving health equity, it is critical that healthcare organizations successfully deploy innovations that both address social needs, provide accessible evidence-based care, and are financially sustainable. Our findings and resultant framework can be used to further examine what ICs lead to successful innovation implementation and improve health equity while also being fiscally sustainable.

## Figures and Tables

**Table 1:** Participating organizations and data sources

	Legal status	∼# patient encounters/month	Number of individuals interviewed and gender (Female-F or Male-M)	Additional data sources
Organization A	Foreign Private Nonprofit Entity With Public Partnerships, Academic Teaching Hospital	21,000	*F* = 1 (one on one interview) *M* = 1 *F* = 1 (small group interview)	Website, annual reports
Organization B	Foreign Private Nonprofit Entity With Public Partnerships, Academic Teaching Hospital	8,400	*F* = 1 *M* = 3 (small group interview)	Website, annual reports
Organization C	Foreign Public-Private Nonprofit Partnership that Earns Revenue	3,000	*F* = 1 *M* = 1 (small group interview)	Website, annual reports
Organization D	Haitian Nonprofit Entity that Earns Revenue	11,000	*M* = 1 *F* = 1 (one on one interviews)	Website, articles in popular press
Organization E	Haitian For Profit Entity with a Not-For-Profit Subsidiary	1,800	*F* = 1 *M* = 1 (small group interview)	Website, annual reports
Organization F	Haitian Nonprofit Entity that Earns Revenue	600	*F* = 1 *F* = 1 (one on one interviews)	Website, articles in popular press

**Source(s):** Authors’ own creation/work

**Table 2:** Summary of study themes

Primary themes	Definition	Examples from study analysis
Product/service innovation	Includes the initiation, implementation, and/or adaptation of core and complementary offerings by an organization, their features, and functionality (Doblin, 2015)	• Team based care delivery • Open medical record systems • Community pharmacies • Specialties, e.g. emergency care services, surgery (and operating rooms), ear, nose, and throat services
Social innovation	Solutions to social problems that are more just, effective, efficient, and/or sustainable than existing solutions and accrue value to society as a whole (Phills *et al.*, 2008)	• Hiring many people from the community • Financial literacy and micro-credit initiatives • Water and sanitation • Educational programs
Business model innovation	Accounts for how the profit structure, i.e. the margin between the assets and fixed cost structure, supports the value proposition, i.e. the product or service that helps customers do more effectively, conveniently, and affordably a job they’ve been trying to do (Hwang and Christensen, 2008)	• Fee for service • Insurance plans • Crowd-sourced community fund for referral care • Government partnerships/collaborations (e.g. with Ministry of Health) • International NGO/Business partnerships/collaborations
Innovation linkage-organizational system behaviors	Includes need identification and opportunity identification behaviors such as searching within the organization, the communities it serves, and the broader health ecosystem for unsolved issues, patient needs, and ideas (Glandon and Counte, 1995; Tan *et al.*, 2005)	• Market need and opportunity identification, e.g. observed weaknesses of healthcare system • Social need and opportunity identification, e.g. proximity/physical access, affordability, malnutrition
Innovation linkage-organizational characteristics	Includes autonomy, prior knowledge, and environmental understanding that may inspire organizational members to innovate despite these perceived constraints	• Desire for autonomy, e.g. focus on self-funding as external/international support decreases • Prior knowledge, e.g. Background in medicine, nursing, agronomy, finance • Flexibility to work with other stakeholders

**Source(s):** Authors’ own creation/work

**Table 3:** Innovation configuration findings

Organization	Category	Health product/Service innovations	Social innovations	Business model innovations	Linkages
Org. A	Healthcare Delivery	Infrastructure: Quasi-Public-Private tertiary referral 300 bed facility. Part of network of facilities that includes 2 hospitals and 10 clinics Services: Inpatient, Outpatient, Women’s Health (reproductive health, maternity ward, pediatric, and NICU), 24/7 Emergency Care, Mental Health, Radiology, Chemotherapy, Laboratory, Surgery, Pharmacy, Rehabilitation, Blood Bank, Community Health Services, HIV and TB clinic Patient clinic card to link patients with their records Continuity of care for chronic condition/long-term communicable disease patients	Apart from an initial 50 HTG/<1 USD registration fee, services are free	Foreign-based, public-private partnership with national government Private donors Partnerships with other healthcare organizations	Market need and opportunity identification: lack of domestic health funding
	Social Determinants of Health	Therapeutic Food via Production Facility	Vocational training Programs including agriculture, literacy, and entrepreneurship Community garden	Partnerships with pharmaceutical companies and pharmaceutical company foundations Job and new market creation	Social need and opportunity identification: e.g. as a underlying problem identified through health services provision such as malnutrition or overall observation of poverty Illustrative quote: *“So, when [a patient comes for care] … we are telling them when they come, they have to pay to stay here; they have to pay the transportation and their food … we are not attracting these people; we are just, you know, keeping them away, not nice, not fair to me … so [Organization A] is trying to [do] whatever they can to cover the fees, allowing us to provide … efficient care to a population -- who have nothing, definitely nothing.”* Organizational Characteristics: Staff with health and agriculture training/knowledge
Org. B	Healthcare Delivery	Infrastructure: Hospital and satellite clinic Services: Holistic care model including Inpatient, Outpatient, Surgery, Maternal Care, NICU, Pediatrics, Elderly Care, Rehabilitation, Infectious and Non-communicable Disease Management, Oral Care, Laboratory Services, Radiology, Residency program and Biomedical Engineering Training program, Vaccination Programs and Community Health Services	Free inpatient care with small fee (150 HTG = ∼ 3 USD) for some outpatient services as free care incentivizes access	Foreign-based, private non-profit model with free services Private donors, NGOs, agencies, and foundation partnerships	Market need and opportunity identification: lack of domestic health funding and Economic return of health initiatives (e.g. vaccinations, surgery)
	Social Determinants of Health	Water and Energy provision from hospital for hospital operations	Women entrepreneurship, microfinance, livestock, and tree farming programs Community farmers market and overall community development Illustrative quote: *“so we actually have a water system … the hospital has invested in the pump and the generators around the system … we just haven’t quite made that jump, you know, from basically sort of free water [that we offer to the community], you know, to, you know, folks actually paying for water where you could take that payment and both pay the staff but reinvest it in, you know, long, you know, more pipeline.”*	Future Ideas: Expand access to water and energy to the municipality and charge a small fee	Market need and opportunity identification: Human and natural resource opportunities (e.g. empowering women entrepreneurs; development of livestock products like chicken/egg farms) Organizational Characteristics: Public Community relationships and opportunity to serve as an advocacy leader
Organization C	Healthcare Delivery	Infrastructure: 5 clinics (3 private, 2 public government primary centers), medical records system Services: Outpatient, Primary Care, Home Visits, Maternal Care and Nutrition, Pharmacy, Laboratory, Vaccination Programs and Community Health Services	Free services at government primary centers, including vaccinations and contraceptives, and free antenatal maternal and child nutrition care at all clinics	Foreign-based, Governmental partnership model: Combination of government, philanthropic donations, and earned revenue	Organizational Characteristics: Previously inflexible approach to NGO support of public clinics
	Social Determinants of Health	Less focus to date	Advocate for NGO support of water and sanitation services	NGO and governmental agency support	Organizational Characteristics: Environmental understanding of Partnership across sectors Illustrative quote: *“I can say that some organizations come with their means and had their own vision and approach on how it intended to work on the ground. When I refer to flexibility, it is we have entered a partnership with [the Ministry of Health] and it may be the case that the approach they wanted to take to achieve their results is not the same way that we see it … Still we need to both understand each other and to figure out a mutually agreeable way to collaborate.”*
Organization D	Healthcare Delivery	Infrastructure: 8 neighborhood hospitals (8/16/24 beds), 20 medical centers, Operating Rooms, and Labs Services: Inpatient, Outpatient, Primary Care, 24/7 Emergency Care, Radiology, Laboratory, Surgery, and Community Pharmacies, Health education, family planning, AIDS prevention, Cholera education, immunization	Facilities in the vicinity of workplaces and neighborhoods Hiring local, community-connected staff Organized sales strategy around social institutions such as churches, schools, scouts etc	Local, Self-Funded Private NGO model Employee Insurance for Manufacturers: $18 USD for 6 months of service at any facility and drug price of $0.60 for any medication Affordable insurance for more vulnerable individuals at affordable rates with a varied range of services based on $1, $5, $10 USD monthly One NGO partnership for marketing and service expansion Future health mutual offering where members of Haitian diaspora can purchase insurance for in-country family members Illustrative quote: *“The idea behind [Organization D] was to offer quality service at a cost communities and people could afford so it was not just offering healthcare services but it was offering quality services at a cost that people could afford. So, this is the reason why I started some kind of HMO where I started building clinics and where I started selling healthcare programs to industries and factories ….”*	Market need and opportunity identification: lack of domestic health funding and lack of private and international support and donations Organizational Characteristics: staff with public health training/knowledge and determined autonomy Illustrative quote: *“I didn’t want to be part of a larger institution; I wanted to be sure that I could secure my own funding to finance my activities.”*
	Social Determinants of Health	Less focus to date (some interest in water and sanitation support)	Less focus	Less focus to date	N/A
Organization E	Healthcare Delivery	Infrastructure: Shared facility and equipment for multiple physicians Services: Ear, Nose and Throat surgical specialty, Ophthalmology care, Radiology services, ENT residency program, Team-based care delivery, Community Health Worker program	Created separate foundation where free or discounted care programs are offered to low-income individuals	Local Healthcare corporation (first in country) Multi-physician private practice (innovative model in Haiti)	Market need and opportunity identification: lack of bank loans to individual physicians; need to increase collaboration between doctors via sharing resources (e.g. equipment) and goodwill Illustrative quote *“The banks don’t like lending to (individual) doctors anyways because the risk of loss is too high and they are right! Financially, they are right. If a group of doctors went to banks with a proposal, the banks can see that within a year the loan can be recovered, they will lend the funds.”* Social need and opportunity identification: response to poverty
	Social Determinants of Health	Less focus to date	Less focus to date	Less focus to date	N/A
Organization F	Healthcare Delivery	Infrastructure: Clinic, Equipment, Laboratory, and medical training for local (future) doctors, nurses, and CHWs who return to community Services: Primary Care, OB/GYN (including deliveries), Pharmacy, Referrals, Community Health Worker Assessments, Immunizations and Visits, School Health Visits, Health Education, Home health care, treatment, and referrals	Facilities in community Supporting training and hiring of community staff Community fund to support referrals for more complex outpatient cases and inpatient care Advocate for Ministry of Health support Community advocates and development committees	Local, Self-Funded Private model with dual Haiti/U.S. NGO status Fee for service (medications included) Ministry of Health provides financial support for some medical staff Foreign partner financial support is community oriented and supports infrastructure needs (e.g. no individual child sponsorships or donated used clothing/items) Clinic “ownership” belongs to founding members in the community, not Org. F	Market need and opportunity identification: Need for care across 10 rural communities, e.g. infantile deaths from unsanitary exposure or malnutrition Organizational Characteristics: Staff with medical and public health training/knowledge and autonomy, specifically aims to shift beneficiary mindset
	Social Determinants of Health	Community Latrines Water filters	Community Meetings and Trainings, Community School, Microcredit Service, Agricultural Loans, Student Scholarships and Loans	Revenue from microcredit and loans supports growth of these services Students from school return to support community School “ownership” belongs to founding members in the community, not Org. F	Social need and opportunity identification: Need and desire for citizen engagement and agency Organizational Characteristics: Willingness for partnership, but within the context of true community engagement Illustrative quote: *They have community meetings upon community meetings to really change the beneficiary mindset to “hey, I have the knowledge, my knowledge counts and [we] are the best change agents for [our] own communities.”*

**Source(s):** Authors’ own creation/work
